# Differences in the Buccolingual Inclinations of Premolars Among Class II Division I Subjects With Different Vertical Facial Patterns

**DOI:** 10.7759/cureus.72667

**Published:** 2024-10-29

**Authors:** Ali B Barri, Bilal F Mahmoud, Ali S Elwan, Radwan A Haffaf, Fadi Khalil

**Affiliations:** 1 Orthodontics, Private Practice, Doha, QAT; 2 Orthodontics and Dentofacial Orthopedics, Tishreen University, Latakia, SYR; 3 Removable Prosthodontics, Tishreen University, Latakia, SYR; 4 Medical Education Program, Syrian Virtual University, Damascus, SYR

**Keywords:** 3d cephalometrics, buccolingual premolar inclination, cbct, class ii, vertical facial pattern

## Abstract

Background: Understanding the attributes of vertical patterns is essential for delivering optimal orthodontic care. Cone-beam computed tomography (CBCT) represents a valuable resource for assessing the buccolingual inclination of the teeth. The present research delves into examining the buccolingual inclination of premolars in nongrowing individuals exhibiting diverse vertical facial patterns.

Methods: CBCT scans of 66 adult patients (31 males and 35 females), mean age 31.6 years (SD = 6.4 years), exhibiting class II division I maxillomandibular relationships, were employed. Participants were categorized into three groups based on linear and angular measurements: normodivergent group (n = 22), hypodivergent group (n = 22), and hyperdivergent group (n = 22). The independent samples t-test and Mann-Whitney U test were conducted to investigate statistical differences in the buccolingual inclination of the premolars among the three vertical patterns.

Results: Statistically significant differences between the three groups were observed in the buccolingual inclinations of both maxillary and mandibular first and second premolars (p < 0.05). The upper right first premolars exhibited a significantly increased buccal inclination in the normodivergent group compared to the hypodivergent group (p < 0.05). In contrast, the two groups had no notable differences in the inclination of the remaining premolars. The upper right and left first premolars exhibited a significantly increased buccal inclination in the hyperdivergent group compared to the hypodivergent group (p < 0.05). In contrast, there were no notable differences in the inclination of the remaining premolars between the two groups. The lower left first premolars exhibited a significantly increased lingual inclination in the hyperdivergent group compared to the normodivergent group (p < 0.05). In contrast, the two groups had no notable differences in the inclination of the remaining premolars. The upper right second premolars exhibited a significantly increased buccal inclination, and the lower left second premolars exhibited greater lingual inclination in the normodivergent group than in the hypodivergent group (p < 0.05). In contrast, there were no notable differences in the inclination of the remaining premolars between the two groups. The upper right and left second premolars exhibited a significantly increased buccal inclination, and the lower left second premolars exhibited greater lingual inclination in the hyperdivergent group than in the hypodivergent group (p < 0.05). In contrast, there were no notable differences in the inclination of the lower right second premolars between the two groups. There were no notable differences in the inclination of the second premolars between the hyperdivergent and normodivergent groups.

Conclusions: In individuals presenting with class II relationships, the buccolingual inclinations of the first and second premolars exhibit similarities and variations across distinct facial patterns. As such, it is imperative for orthodontic practitioners to exercise meticulous consideration of these nuances throughout treatment.

## Introduction

The vertical facial type and orthodontic intervention are closely connected, with distinct variations in alveolar bone, muscle, and malocclusion observed based on the vertical facial type. These differences have significant implications for treatment strategization [1]. Orthodontic treatment planning necessitates a thorough evaluation of individual patient attributes, encompassing age, gender, and ethnicity, alongside a comprehensive assessment of the facial pattern and its associated clinical characteristics, which play a pivotal role [2]. The facial pattern significantly impacts the determination of anchorage type, the prognostication of craniofacial development, and the overarching objectives of treatment [3]. These objectives encompass harmonizing dental positioning within the dental arches, rectifying malocclusion to attain a durable dental occlusion, and enhancing functional and esthetic outcomes [4]. To attain these goals, it is necessary for the maxillary dentoalveolar bone to have adequate transverse width and for the posterior teeth to display ideal buccolingual angulations [5]. From a frontal perspective, the convexity of the maxillary posterior occlusal surfaces and the concavity of the mandibular posterior occlusal surfaces contribute to the curvature of the occlusal plane, which typically assumes a curved form, and this occlusal curve in the coronal plane is referred to as Wilson's curve [6]. The inherent curve of the teeth is believed to enable the posterior teeth to align with the direction and inward force exerted by the medial pterygoid muscle upon contraction [6]. This alignment optimizes the teeth's ability to withstand chewing forces, promotes efficient food processing, and guarantees effective contact between the tooth cusps [6,7]. Thus, it is essential to investigate the dental inclination that contributes to the development of Wilson's arch.

Multiple research studies focusing on the buccolingual inclination of the posterior teeth have commonly categorized their subjects into groups according to sagittal or vertical skeletal characteristics [4,5,8,9]. In the extant literature, the predominant focus has been on investigations of the first and second molars. Like all other teeth, the premolars play a crucial role in effective chewing by withstanding occlusal pressures and are significant in preserving the vertical dimension of facial structure. Hence, premolars play a dual role in functionality and facial esthetics [1]. Numerous studies have examined buccolingual inclination through the utilization of plaster models; however, there are inherent limitations associated with their application [10].

In recent years, there has been a notable advancement in head and neck imaging with the introduction of cone-beam computed tomography (CBCT) devices. Specifically designed for maxillofacial diagnostics, these devices have proven to be cost-effective and have significantly reduced radiation exposure for patients [11]. Their capability to produce high-resolution images with isotropic voxel matrices has made them particularly suitable for imaging the craniofacial area, thereby enhancing the precision of treatment planning [11]. Moreover, CBCT technology has been instrumental in accurately determining the spatial relationship between teeth and anatomical landmarks [12].

The primary objective of this research was to determine the buccolingual inclination of the premolars utilizing CBCT among individuals classified as class II division I with varying vertical facial patterns. These findings are anticipated to offer valuable insights that can enhance orthodontic treatment planning efficacy during orthodontic procedures.

## Materials and methods

The study was approved by the Institutional Review Board of Tishreen University under approval no. 4338. The retrospective study utilized CBCT scans acquired from 2014 to 2022 from the Faculty of Dentistry, Tishreen University. Eligible participants were required to meet specific inclusion criteria, which encompassed an age range of 18 to 40 years and classification as class II division I individuals exhibiting an an angle formed by points A, N, and B that exceeds 4° [13]. Furthermore, it was essential that they did not have any diagnosed systemic diseases, craniofacial dysmorphologies, impacted or missing teeth, periodontal disease, facial asymmetries exceeding 2 mm from the midsagittal plane (ME), or conditions such as cleft lip or palate. Additionally, the incisor angle relative to the Frankfurt horizontal plane had to be 110° or greater [14]. Angular and linear measurements were employed to determine the vertical growth pattern. The sella-nasion to gonion-menton (S-N/Go-Me) angle was utilized as a determinant of vertical divergence: S-N/Go-Me angles below 30.5° denoted hypodivergence, those between 30.5° and 35.5° represented normodivergence, and angles exceeding 35.5° indicated hyperdivergence [15]. The S-Go/N-Me ratio was also utilized: ratios below 61% implied hyperdivergence, ratios between 61% and 69% represented normodivergence, and ratios exceeding 69% denoted hypodivergence [16]. Subjects who did not meet these criteria were excluded from the study. CBCT scans were obtained employing the following parameters: 85 kVp, 15 mA, exposure time 40 seconds, focal spot size 3.3 mm, and voxel resolution 0.090 mm. The resulting data were generated utilizing a Scanora 3D CBCT Sordex unit (Tuusula, Finland) and preserved in Digital Imaging and Communication in Medicine format.

A power analysis was executed with the parameters α = 0.05, power = 0.8, and effect size = 0.8, yielding a required sample size of n = 66 to attain adequate statistical power. Table 1 presents the distribution of patients across the three groups and statistical data for the measurements that formed the basis for group classification.

**Table 1:** The number of females and males in each group. Statistics for the measurements that were the basis for classification between the groups are displayed Values are presented as N (%) or mean ± standard deviation ANB: the angle formed between points A, N, and B; S N-Go Me: the angle formed between the line (S-N) and the line (Go-Me); S-Go/N-Me: the ratio of the length of the line segment S-Go to the length of the line segment N-Me; U1: the axis of the upper central incisors; U1-FH: the angle formed between the axis of the maxillary central incisors and the Frankfort horizontal plane

Basis for classification	Hyperdivergent	Normodivergent	Hypodivergent
Gender			
Male	8 (36.4%)	8 (36.4%)	15 (68.3%)
Female	14 (63.6%)	14 (63.6%)	7 (31.8%)
ANB (°)	7.41° ± 2.29°	6.23° ± 1.84°	6.42° ± 1.23°
S N-GO Me (°)	42.27° ± 4.58°	33.66° ± 1.75°	27.31° ± 2.82°
S-Go/N-Me (%)	57% ± 2.45%	65% ± 3.22%	71% ± 1.56%
U1-FH (°)	117.77° ± 2.34°	114.56° ± 1.75°	115.89° ± 1.43°

Analysis of Radiographs Using OnDemand program version 1.0.10.7462 (Cybermed Inc., Seoul, Korea) was employed for radiographic analysis. The nasion (N) was designated as the origin of the three coordinate planes (X, Y, Z). The orbital plane was established by the right and left orbitales (Or) and the left portion (Po). The horizontal plane (X) was defined as the plane parallel to the orbital plane and passing through N. The ME (Y) was defined as the plane perpendicular to the horizontal plane, passing through N and the anterior nasal spines. Finally, the frontal plane (Z) was perpendicular to both the horizontal and MEs, passing through N [8]. Subsequently, a customized analysis was created within the program's 3D Ceph section, incorporating code to precisely identify the anatomical landmarks listed in Table 2.

**Table 2:** The landmarks that were determined for each cone-beam computed tomography scan N: nasion; S: sella; Po: porion; ANS: anterior nasal spine; Me: menton; Go: gonion; L Or: left orbitale; R Or: right orbitale

Landmark	Definition of the landmark
N	The junction between the nasal and frontonasal sutures
S	The center of the sella turcica on the midsagittal plane
Po	The most superior point on the upper rim of the external auditory meatus
A	The deepest point between the anterior nasal spine and prosthion at the midsagittal plane
B	The deepest point between the pogonion and the alveolus of the lower incisors on the midsagittal plane
ANS	The most anterior point on the floor of the nose
Me	The lowermost point on the symphysis menti on the midsagittal plane
Go	The midway between the lowermost point on the posterior border of the ramus and the most posterior point on the lower border of the mandible
R Or	The most inferior point on the lower rim of the right orbit
L Or	The most inferior point on the lower rim of the left orbit
Crown point	A point within the level of the tooth crown that was used to determine the longitudinal axis of the premolar
Root point	A point within the plane of the root apex that was used to determine the longitudinal axis of the premolar

**Figure 1:**
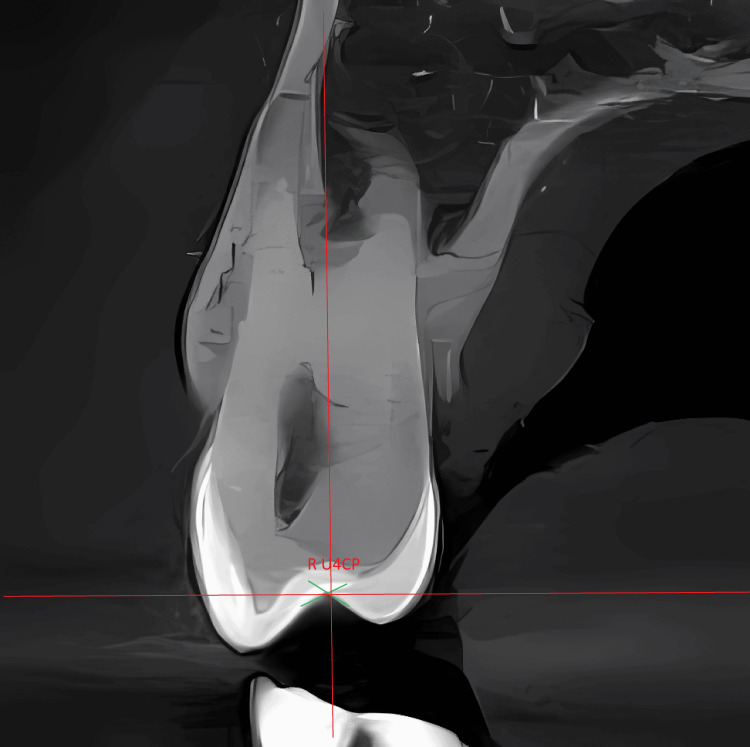
Point representing the central fossa R U4CP: right maxillary first premolar crown point

**Figure 2:**
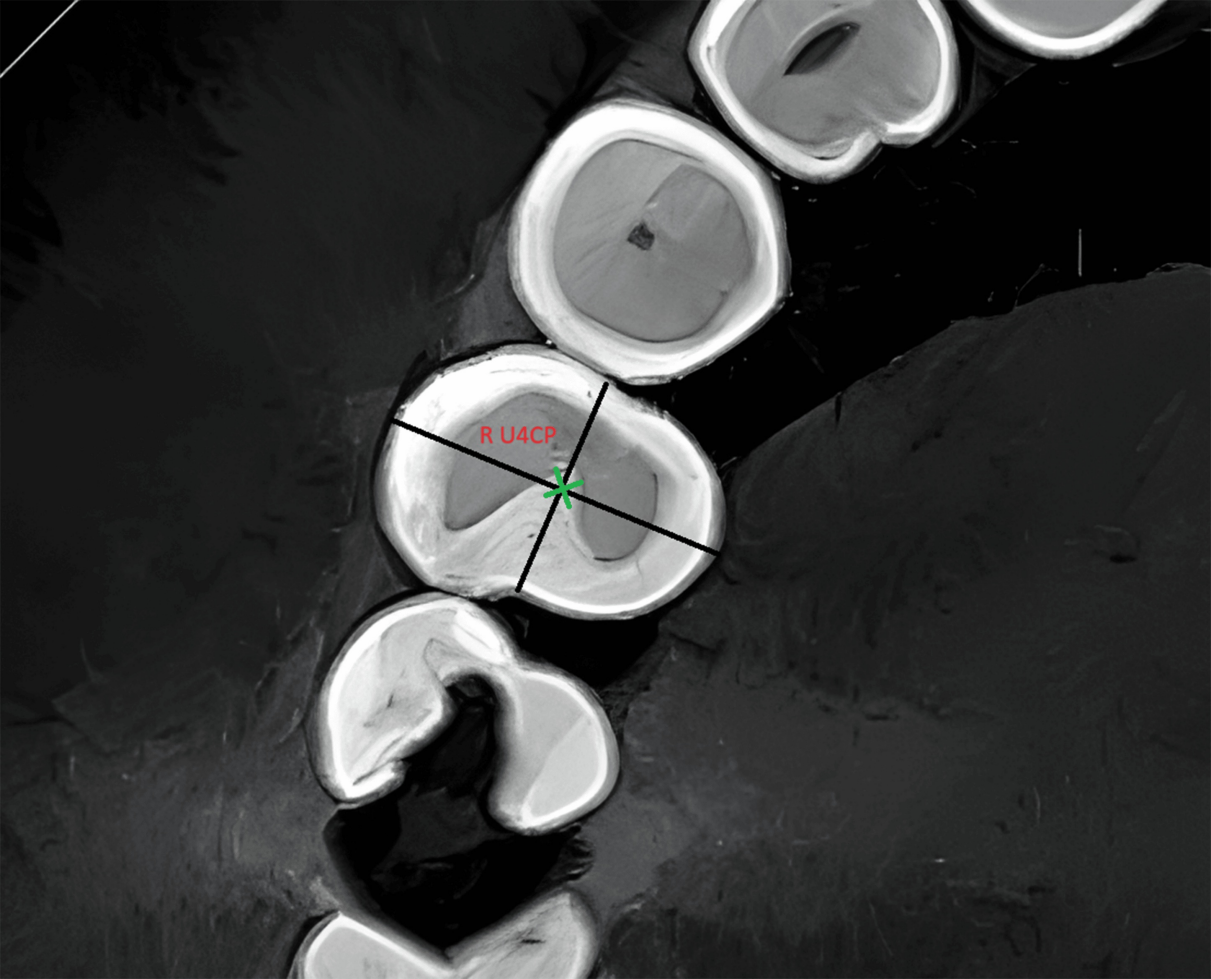
Determination of the crown point R U4CP: right maxillary first premolar crown point

For all premolars, the crown point was determined through the following: 1) in the frontal plane, identify a point that represents the central fossa of the premolar (Figure 1), and 2) in the occlusal plane, two lines representing the two diagonals were determined at a level corresponding to the depth of the central fossa. The crown point was determined by the intersection point of the two diagonals that were established earlier (Figure 2).

**Figure 3:**
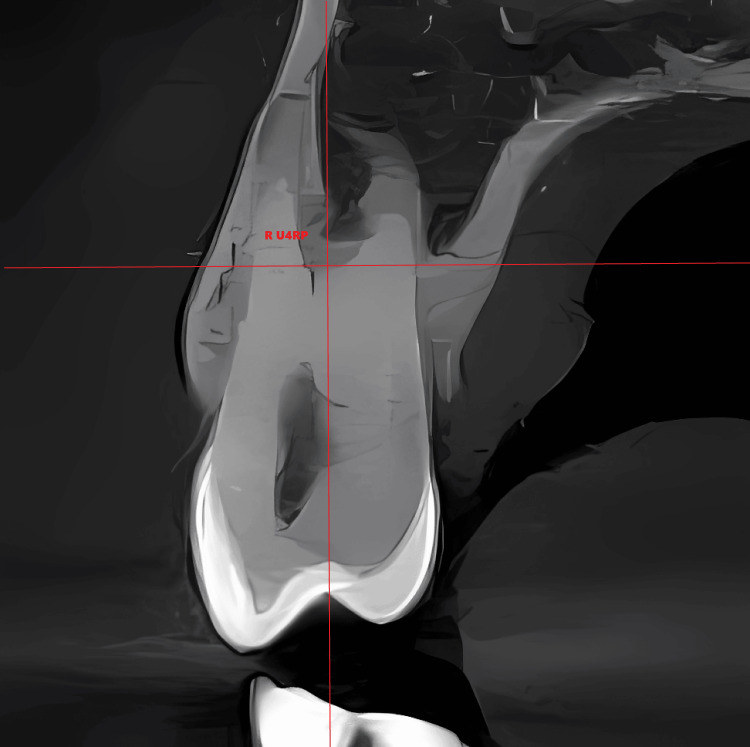
The axial axis passing through the premolar bifurcation R U4RP: right maxillary first premolar root point

**Figure 4:**
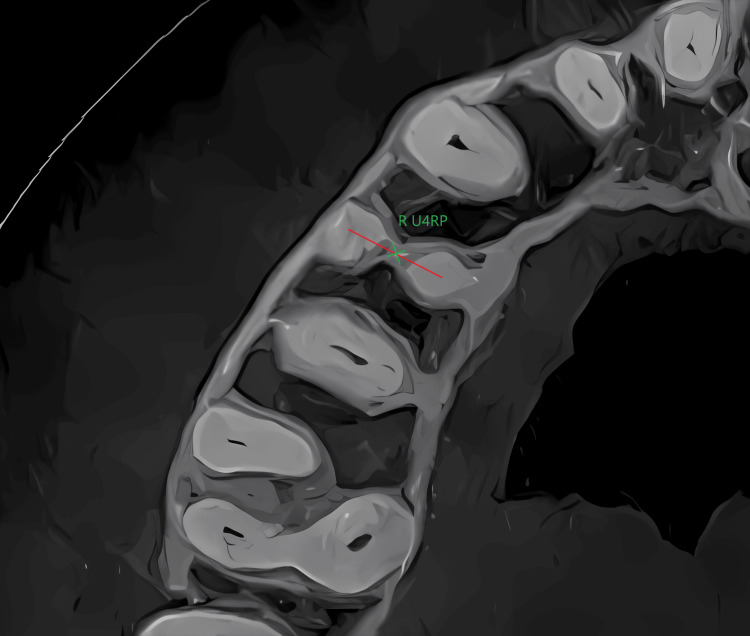
Determination of the root point R U4RP: right maxillary first premolar root point

The root point was determined through the following: 1) in the frontal plane, the identification of the root junction point was conducted for teeth exhibiting two roots. In cases where the tooth presented a single root, the apex of the tooth was utilized as a reference point. Specifically, when the angle of apical curvature exceeded 10°, the point immediately preceding the curvature was selected for analysis (Figure 3), and 2) in the occlusal plane, in the case of a two-rooted tooth, the midpoint between the two root canals was determined. In the case of a single-rooted tooth, the apex or point preceding the curvature was determined (Figure 4).

**Figure 5:**
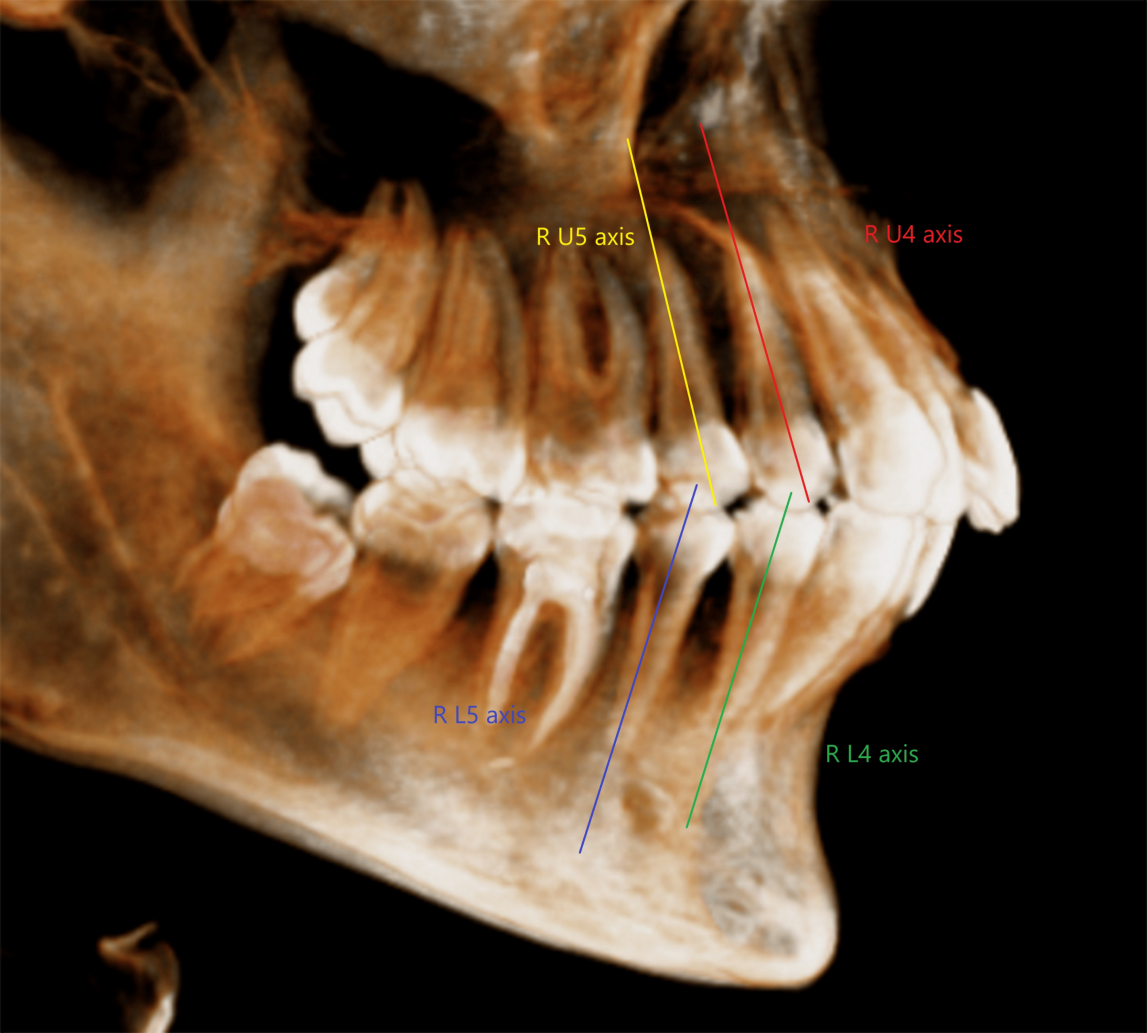
Premolars axes R U4: right maxillary first premolar; R U5: right maxillary second premolar; R L4: right mandibular first premolar; R L5: right mandibular second premolar

The premolar axis was established by the line connecting the crown point and the root point (Figure 5).

Reference line and premolar inclination

The reference line was formed by the line connecting the right and left orbital points (Or-Or). The buccolingual inclination of the premolar was measured relative to this reference line as follows.

**Figure 6:**
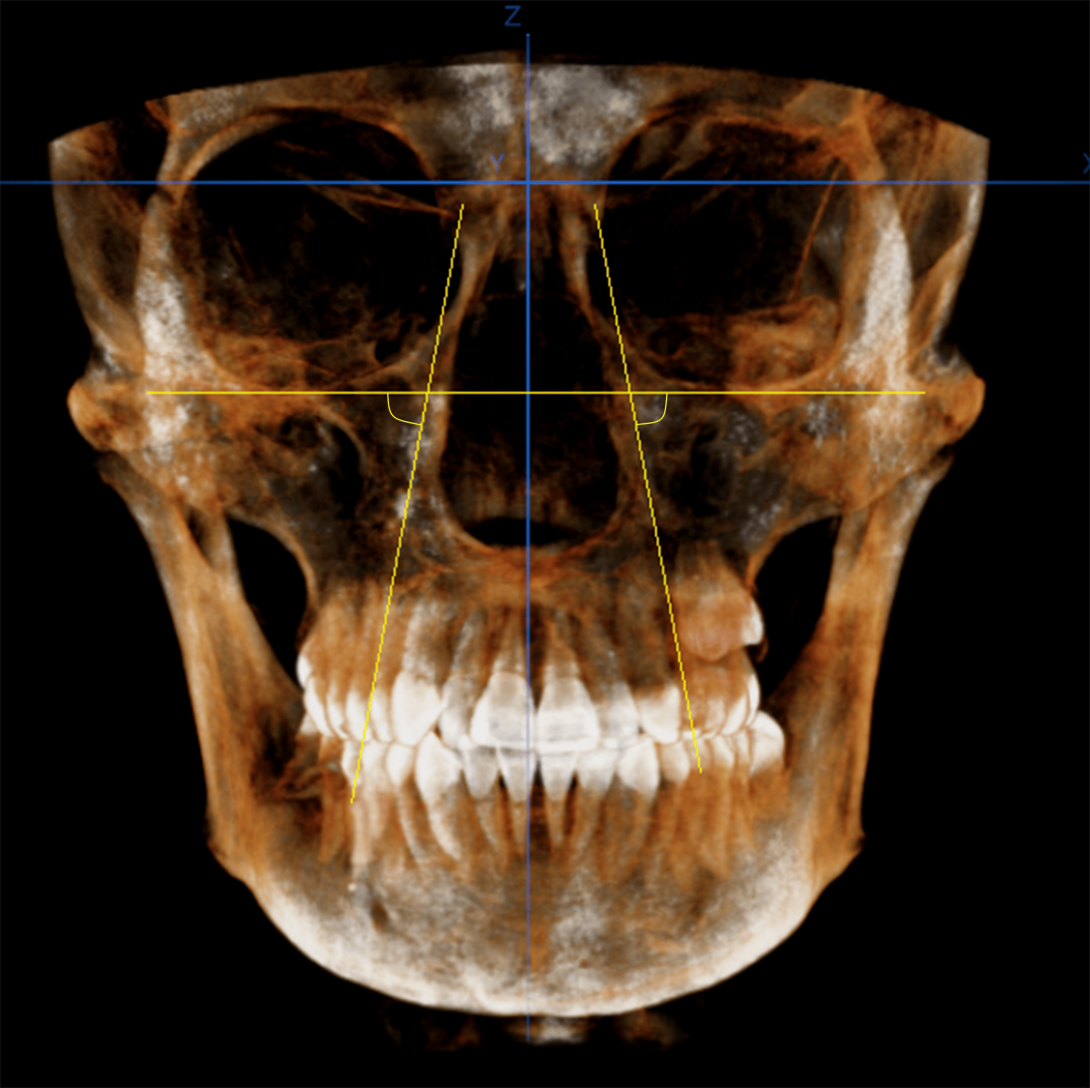
Measurement of the right and left maxillary first premolar buccolingual inclinations Yellow horizontal line: the line orbitale-orbitale; vertical yellow lines: maxillary first premolars axes; blue lines: cone-beam computed tomography orientation planes

1) A projection was created for the premolar axis at the frontal plane.

2) A projection of the Or-Or line was created at the frontal plane.

3) The angle formed by the projections of the previous two lines was measured and was taken as the value of the buccolingual inclination of the premolar (Figure 6).

Statistical analysis

Statistical Package for the Social Sciences version 28 (IBM Corp., Armonk, NY) was used to conduct the statistical analyses. Descriptive statistics such as means and standard deviations (SDs) were calculated. The Shapiro-Wilk test was conducted to evaluate the data normality. All variables, with the exception of three (mandibular left second premolar and maxillary right second premolar in the normodivergent group, and maxillary left first premolar in the hypodivergent group), were normally distributed. The independent samples t-test and Mann-Whitney U test were conducted for group comparison. Statistical significance was evaluated at a p value threshold of 0.05, corresponding to a 95% confidence interval.

One week after the initial data acquisition, the researcher B.F.M. obtained a subsequent set of measurements to ensure intraobserver reliability. This reliability was evaluated by computing intraclass correlation coefficients, which serve as a quantitative measure of any systematic measurement error.

## Results

This study employed 66 CBCT scans, comprising a sample of 31 males (46.96%) and 35 females (53.03%), with 22 subjects in each of the three groups (hypodivergent, normodivergent, and hyperdivergent). The range of the intraclass correlation coefficients was 0.867-0.945, demonstrating high intraobserver reliability during repeated measurements. The values of buccolingual inclination of the maxillary and mandibular premolars are presented in Tables 3-12.

**Table 3:** Values of buccolingual inclination and the results of the independent samples t-test to study the differences between the average angle of the first premolar between the hyperdivergent and normodivergent groups Values are presented as number (N) or mean ± standard deviation U R4: maxillary right first premolar; U L4: maxillary left first premolar; L R4: mandibular right first premolar; L L4: mandibular left first premolar p < 0.05 was considered significant

Tooth	Hyperdivergent	Normodivergent	Mean difference	t value	p value
U R4	86.60° ± 5.17°	88.89° ± 6.81°	2.28	1.254875	0.216
U L4	85.30° ± 5.46°	87.96° ± 3.64°	2.65	1.897659	0.065
L R4	87.27° ± 4.87°	88.99° ± 3.80°	1.71	1.303251	0.20
L L4	90.24° ± 3.97°	87.89° ± 2.65°	2.34	-2.305632	0.026

**Table 4:** Values of buccolingual inclination and the results of the independent samples t-test to study the differences between the average angle of the first premolar between the hypodivergent and normodivergent groups Values are presented as number (N) or mean ± standard deviation U R4: maxillary right first premolar; L R4: mandibular right first premolar; L L4: mandibular left first premolar p < 0.05 was considered significant

Tooth	Hypodivergent	Normodivergent	Mean difference	t value	p value
U R4	93.36° ± 3.36°	88.89° ± 6.81°	4.47	2.762053	0.008
L R4	86.99° ± 3.80°	88.99° ± 3.80°	-2	-1.748943	0.088
L L4	88.36° ± 3.86°	87.89° ± 2.65°	0.47	0.473925	0.638

**Table 5:** Values of buccolingual inclination and the results of the Mann-Whitney U test to study the differences between the average ranks of the first premolar angle between the hypodivergent and normodivergent groups Values are presented as number (N) or mean ± standard deviation U L4: maxillary left first premolar p < 0.05 was considered significant

Tooth	Hypodivergent	Normodivergent	Mann-Whitney U value	z value	p value
U L4	90.54° ± 4.73°	87.96° ± 3.64°	170.00	-1.690446	0.091

**Table 6:** Values of buccolingual inclination and the results of the independent samples t-test to study the differences between the average angle of the first premolar between the hyperdivergent and hypodivergent groups Values are presented as number (N) or mean ± standard deviation U R4: maxillary right first premolar; L R4: mandibular right first premolar; L L4: mandibular left first premolar p < 0.05 was considered significant

Tooth	Hypodivergent	Hyperdivergent	Mean difference	t value	p value
U R4	93.36° ± 3.36°	86.60° ± 4.69°	6.76	5.135529	0.000
L R4	86.99° ± 3.80°	87.27° ± 2.75°	-0.28	-0.217545	0.82
L L4	88.36° ± 3.86°	90.24° ± 2.75°	1.87	-1.586790	0.12

**Table 7:** Values of buccolingual inclination and the results of the Mann-Whitney U test to study the differences between the average ranks of the first premolar angle between the hypodivergent and hyperdivergent groups Values are presented as number (N) or mean ± standard deviation U L4: maxillary left first premolar p < 0.05 was considered significant

Tooth	Hypodivergent	Hyperdivergent	Mann-Whitney U value	z value	p value
U L4	90.54° ± 4.73°	85.30° ± 5.46°	118.00	-2.911119	0.004

**Table 8:** Values of buccolingual inclination and the results of the independent samples t-test to study the differences between the average angle of the second premolar between the hyperdivergent and normodivergent groups Values are presented as number (N) or mean ± standard deviation U L5: maxillary left second premolar; L R5: mandibular right second premolar p < 0.05 was considered significant

Tooth	Hyperdivergent	Normodivergent	Mean difference	t value	p value
U L5	86.76° ± 5.50°	89.35° ± 4.67°	2.58	1.678029	0.101
L R5	92.85° ± 4.64°	95.39° ± 4.17°	2.54	1.913904	0.062

**Table 9:** Values of buccolingual inclination and the results of the Mann-Whitney U test to study the differences between the average ranks of the second premolar angle between the hyperdivergent and normodivergent groups Values are presented as number (N) or mean ± standard deviation U R5: maxillary right second premolar; L L5: mandibular left second premolar p < 0.05 was considered significant

Tooth	Hyperdivergent	Normodivergent	Mann-Whitney U value	z value	p value
U R5	87.04° ± 5.80°	88.90° ± 4.61°	183.00	-1.385178	0.166
L L5	95.75° ± 3.88°	94.45° ± 6.41°	241.00	-0.023478	0.981

**Table 10:** Values of buccolingual inclination and the results of the independent samples t-test to study the differences between the average angle of the second premolar between the hypodivergent and normodivergent groups Values are presented as number (N) or mean ± standard deviation U L5: maxillary left second premolar; L R5: mandibular right second premolar p < 0.05 was considered significant

Tooth	Hypodivergent	Normodivergent	Mean difference	t value	p value
U L5	91.19° ± 6.18°	89.35° ± 4.67°	1.84	1.116599	0.271
L R5	93.12° ± 4.31°	95.39° ± 4.17°	2.27	-1.773419	0.083

**Table 11:** Values of buccolingual inclination and the results of the Mann-Whitney U test to study the differences between the average ranks of the second premolar angle between the hypodivergent and normodivergent groups Values are presented as number (N) or mean ± standard deviation U R5: maxillary right second premolar; L L5: mandibular left second premolar p < 0.05 was considered significant

Tooth	Hypodivergent	Normodivergent	Mann-Whitney U value	z value	p value
U R5	92.23° ± 4.07°	88.90° ± 4.61°	144.50	-2.289226	0.022
L L5	92.65° ± 3.11°	94.45° ± 6.41°	117.00	-2.935113	0.003

**Table 12:** Values of buccolingual inclination and the results of the independent samples t-test to study the differences between the average angle of the second premolar between the hypodivergent and hyperdivergent groups Values are presented as number (N) or mean ± standard deviation U R5: maxillary right second premolar; U L5: maxillary left second premolar; L R5: mandibular right second premolar; L L5: mandibular left second premolar p < 0.05 was considered significant

Tooth	Hypodivergent	Hyperdivergent	Mean difference	t value	p value
U R5	92.23° ± 4.07°	87.04° ± 5.80°	5.18	3.429471	0.001
U L5	91.19° ± 6.18°	86.76° ± 5.50°	4.42	2.509727	0.016
L R5	93.12° ± 4.31°	92.85° ± 4.64°	0.27	0.204811	0.839
L L5	92.65° ± 3.11°	95.75° ± 3.88°	3.10	-2.922456	0.006

Results pertaining to first premolars

Statistical analysis indicated that there was no significant difference in the buccolingual inclinations of the first premolars between the normodivergent and hyperdivergent groups, except for the left mandibular first premolar group (p < 0.05). The mandibular left first premolars exhibited a significantly increased lingual inclination in the hyperdivergent group compared to the normodivergent group (Table 3).

Statistically significant differences were observed between the hypodivergent group and the other groups. These differences were evident in the maxillary right and left premolars (p < 0.05). The maxillary right first premolars exhibited a significantly increased buccal inclination in the normodivergent group compared to the hypodivergent group. The maxillary right and left first premolars exhibited a significantly increased buccal inclination in the hyperdivergent group compared to the hypodivergent group (Tables 4-7).

Results pertaining to second premolars

Statistical analysis indicated no significant difference in the buccolingual inclinations of the second premolars between the normodivergent and hyperdivergent groups (p > 0.05) (Tables 8, 9). Statistically significant differences were identified between the hypodivergent group and the other groups. These differences were particularly notable in the maxillary right, maxillary left, and mandibular left second premolars when compared with the hyperdivergent group, as well as in the maxillary right and mandibular left premolars when compared with the normodivergent group (p < 0.05). The maxillary right and left second premolars exhibited a significantly increased buccal inclination, and the mandibular left second premolars exhibited greater lingual inclination in the hyperdivergent group than in the hypodivergent group. The maxillary right second premolars exhibited a significantly increased buccal inclination, and the mandibular left second premolars exhibited greater lingual inclination in the normodivergent group than in the hypodivergent group (Tables 10-12).

## Discussion

The incorporation of CBCT into both general and specialized dental practices has resulted in a continuous rise in its application across various clinical fields, including orthodontics, implantology, temporomandibular joint imaging, and maxillofacial surgery. This trend can be attributed to the superior accuracy, high resolution, and dependable nature of CBCT technology [17].

The present study incorporated CBCT scans from patients aged between 18 and 40, acknowledging that teeth inclination may change throughout the developmental growth phase. A novel methodology was implemented to ascertain the longitudinal axis of the premolars, with the objective of establishing a three-dimensional axis. This approach marks a departure from the previously utilized two-dimensional longitudinal axis typically applied in the context of three-dimensional structures.

Prior investigations have primarily focused on the buccolingual inclination of first or second molars categorized by skeletal classifications or facial types. However, these studies have overlooked the role of premolars, which are integral to comprehensive orthodontic treatments. A study by Duan et al. demonstrated that the apical buccal removable space within the mandibular first premolar is significantly larger in individuals with a short facial type compared to those with long and normal facial types [1]. These findings imply that the application of torque to the mandibular first premolar may pose an increased risk for individuals with long and normal facial types, as it could lead to the root making contact with the buccal cortex, thereby potentially resulting in detrimental outcomes.

The relationship between sagittal skeletal discrepancies and molar inclinations has been thoroughly examined in existing scientific research, alongside the impact of vertical growth patterns on the positioning of the mandibular first premolars. Therefore, the present study sought to clarify the inclinations of premolars in class II division I individuals presenting with hypodivergent, hyperdivergent, or normodivergent vertical growth patterns. The findings revealed notable discrepancies between the groups. Specifically, the maxillary right first premolars exhibited a significantly increased buccal inclination in the normodivergent group compared to the hypodivergent group. In contrast, there were no notable differences in the inclination of the remaining premolars between the two groups. The maxillary right and left first premolars exhibited a significantly increased buccal inclination in the hyperdivergent group compared to the hypodivergent group. In contrast, the two groups had no notable differences in the inclination of the remaining premolars. The mandibular left first premolars exhibited a significantly increased lingual inclination in the hyperdivergent group compared to the normodivergent group. In contrast, the two groups had no notable differences in the inclination of the remaining premolars.

The maxillary right second premolars exhibited a significantly increased buccal inclination, and the mandibular left second premolars exhibited greater lingual inclination in the normodivergent group than in the hypodivergent group. In contrast, there were no notable differences in the inclination of the remaining premolars between the two groups.

The maxillary right and left second premolars exhibited a significantly increased buccal inclination, and the mandibular left second premolars exhibited greater lingual inclination in the hyperdivergent group than in the hypodivergent group. In contrast, there were no notable differences in the inclination of the mandibular right second premolars between the two groups. There were no notable differences in the inclination of the second premolars between the hyperdivergent and normodivergent groups.

The discrepancies observed in premolar inclination may be accounted for by the broader dental arch present in the hypodivergent group, which necessitates a buccal inclination of the mandibular premolars and a lingual inclination of the maxillary premolars to achieve proper occlusion. Furthermore, this divergence may stem from the differential distribution of muscular tension, both in magnitude and orientation, between the right and left sides of the jaw. Research conducted by Nishi et al. has demonstrated a notable difference in temporalis muscle activity between the left and right sides in individuals with class II malocclusion [18]. Additionally, preliminary findings by Al Zubaidi et al. suggest that abnormal muscular function may impart varying pressures on different regions of the mandible, potentially leading to diverse forms of dentoalveolar underdevelopment [19]. Alternatively, this phenomenon could also arise from variations in the thickness of cortical bone surrounding the mandibular molars.

Equipped with an understanding of the natural inclinations of teeth, clinicians have the capability to effectively adjust these inclinations through the implementation of suitable and physiologically appropriate alignment techniques. The constraints of this study are characterized by the singular assessment of class II division I subjects. Subsequent investigations ought to prioritize the distinct analysis of sagittal and vertical traits to enhance the overall comprehension of variations in dental alignment among normodivergent, hyperdivergent, and hypodivergent individuals across class I, class II, and class III malocclusions. Furthermore, it is essential to correlate premolar inclination with the severity of the malocclusion.

## Conclusions

Statistically significant difference was found between the hyperdivergent, normodivergent and hypodivergent groups in terms of buccolingual premolar inclinations. The premolars exhibited varying degrees of buccal and lingual inclinations, occasionally displaying excessive buccal inclinations and at other times demonstrating pronounced lingual inclinations. This variability suggests significant differences in buccolingual inclination across different facial types. Therefore, it is essential to consider these variations when formulating treatment plans that align with the physiological characteristics of the bone and surrounding tissues.
